# Aldehyde Dehydrogenase Gene Superfamily in *Populus*: Organization and Expression Divergence between Paralogous Gene Pairs

**DOI:** 10.1371/journal.pone.0124669

**Published:** 2015-04-24

**Authors:** Feng-Xia Tian, Jian-Lei Zang, Tan Wang, Yu-Li Xie, Jin Zhang, Jian-Jun Hu

**Affiliations:** 1 College of Life Science and Technology, Nanyang Normal University, Nanyang, Henan, 473061, China; 2 State Key Laboratory of Tree Genetics and Breeding, Key Laboratory of Tree Breeding and Cultivation of the State Forestry Administration, Research Institute of Forestry, Chinese Academy of Forestry, Beijing, 100091, China; 3 Research Institute of Wood Industry, Chinese Academy of Forestry, Beijing, 100091, China; University of Iceland, ICELAND

## Abstract

Aldehyde dehydrogenases (ALDHs) constitute a superfamily of NAD(P)^+^-dependent enzymes that catalyze the irreversible oxidation of a wide range of reactive aldehydes to their corresponding nontoxic carboxylic acids. ALDHs have been studied in many organisms from bacteria to mammals; however, no systematic analyses incorporating genome organization, gene structure, expression profiles, and *cis*-acting elements have been conducted in the model tree species *Populus trichocarpa* thus far. In this study, a comprehensive analysis of the *Populus ALDH* gene superfamily was performed. A total of 26 *Populus ALDH* genes were found to be distributed across 12 chromosomes. Genomic organization analysis indicated that purifying selection may have played a pivotal role in the retention and maintenance of *PtALDH* gene families. The exon-intron organizations of *PtALDHs* were highly conserved within the same family, suggesting that the members of the same family also may have conserved functionalities. Microarray data and qRT-PCR analysis indicated that most *PtALDH*s had distinct tissue-specific expression patterns. The specificity of *cis*-acting elements in the promoter regions of the *PtALDHs* and the divergence of expression patterns between nine paralogous *PtALDH* gene pairs suggested that gene duplications may have freed the duplicate genes from the functional constraints. The expression levels of some *ALDHs* were up- or down-regulated by various abiotic stresses, implying that the products of these genes may be involved in the adaptation of *Populus* to abiotic stresses. Overall, the data obtained from our investigation contribute to a better understanding of the complexity of the *Populus ALDH* gene superfamily and provide insights into the function and evolution of *ALDH* gene families in vascular plants.

## Introduction

Endogenous aldehydes are intermediates or byproducts in a range of fundamental biochemical pathways that are generated during the metabolism of carbohydrates, vitamins, steroids, amino acids, and lipids [1, 2]. When produced in excessive amounts, these aldehydes can have detrimental effects on cellular metabolism because of their chemical reactivity [3–5]. Therefore, cellular levels of aldehydes need to be regulated to maintain normal developmental processes. One of the major detoxification pathways of aldehyde molecules involves the oxidation of the carbonyl groups to carboxylic acids by NAD(P)^+^-dependent enzymes aldehyde dehydrogenases (ALDHs; enzyme class EC: 1.2.1.3) [1–3, 6].

ALDHs are found in both prokaryotes and eukaryotes [2, 3, 6–8]. The ALDH Gene Nomenclature Committee has established specific criteria for cataloguing deduced ALDH protein sequences [6]; namely, protein sequences that share more than 40% identity with other ALDH sequences compose a family, and sequences that share more than 60% identity compose a subfamily. ALDH protein sequences that share less than 40% identity would form a new family. Previous classifications of the *ALDH* gene superfamily in eukaryotes have identified 24 protein families based on sequence identity [7–10]. Among the 24 ALDH families, 14 (ALDH2, ALDH3, ALDH5, ALDH6, ALDH7, ALDH10, ALDH11, ALDH12, ALDH18, ALDH19, ALDH21, ALDH22, ALDH23 and ALDH24) contain members from plant species and seven (ALDH11, ALDH12, ALDH19, ALDH21, ALDH22, ALDH23 and ALDH24) are unique to plants [10].

Of the plant *ALDHs* that have been characterized to date, the majority have been implicated in diverse pathways and appear to play crucial roles in plant growth and development. For example, the maize *ALDH2* gene *rf2* is required for male fertility [11] and the rice *ALDH7* is essential for seed maturation and viability [12]. Many of the plant *ALDHs* are responsive to various environmental stresses, including dehydration, high salinity, heat, water logging, oxidative stress, and heavy metals, suggesting possible roles for these genes in improving stress tolerance [13–15]. Several studies have found that overexpression of some plant *ALDHs* enhanced plant tolerance to diverse types of abiotic and biotic stresses [13–17]. Most of these studies have been performed in model species such as *Arabidopsis* [8] and rice [18], and, until now, little attention has been paid to woody species like *Populus*.

During plant evolution, gene families have undergone copy number selection via duplications, transpositions and/or deletions [19]. Gene duplication and subsequent gene retention or loss (fractionation) are often attributed to recent and/or ancient whole genome polyploidy events, for example, at the origin of seed plants and angiosperms [20]. Whole-genome duplications can buffer gene functions by increasing genetic redundancy and hence contribute to sub- or neo-functionalization, which can drive genetic innovation [21]. For example, paralogous genes derived from a whole-genome duplications that encode structurally similar enzymes have been shown to evolve towards extended substrate specificities or to catalyze novel reactions, whereas the ancestral gene retains its original function [22].

*Populus* species are perennial trees that frequently undergo seasonal variations and various environmental stresses. The completion of the *Populus trichocarpa* genome sequencing project in 2006 made *P*. *trichocarpa* a suitable model for woody plants [23]. An analysis of the *P*. *trichocarpa* genome found that a whole-genome duplication event may be occurred recently (in evolutionary terms) in the stem lineage of the Salicaceae family, about 60 to 65 million years ago, in addition to another, much more ancient large-scale duplication event shared by *Populus* and *Arabidopsis* [23]. The complex history of genome duplications and chromosomal rearrangements in *Populus* provide an opportunity to study gene family expansion patterns over the course of genome evolution [24]. In a previous study, Brocker et al. [25] identified 26 *Populus ALDHs* based on the *P*. *trichocarpa* genome V2.2; however, no systematic analyses of the evolution and expression patterns were analyzed. To determine the structure-function relationship of the *ALDHs* in *Populus*, we performed detailed systematic analyses of the genome organization, gene structure, expression compendium, and *cis*-acting elements in *P*. *trichocarpa*. In this study, we report comprehensive phylogenetic and evolutionary analyses of the 26 members of the *ALDH* superfamily in *Populus*, including their expression profiles in different tissues and their responses under various abiotic stresses. The specificity of *cis*-acting elements between paralogous *Populus ALDH* gene pairs were applied to investigate the divergence of their expression patterns to help understood how paralogous genes play different roles in various biological processes and stress responses. Our results may provide the insights to further investigate the functions of the *ALDHs* in *Populus* species.

## Materials and Methods

### Characteristics of *Populus ALDH* genes

A previous study identified 26 *Populus ALDH* genes based on the *P*. *trichocarpa* genome V2.2 [25]. We also checked the latest *P*. *trichocarpa* genome V3.0 by BLASTP and no more ALDH members were identified. Total of 26 *Populus ALDHs* were named according to Brocker et al. and their coding sequences were download from *P*. *trichocarpa* genome V3.0 (http://phytozome.jgi.doe.gov/pz/portal.html#!info?alias=Org_Ptrichocarpa). To characterize the members of *Populus ALDH* superfamily, WoLF PSORT (http://wolfpsort.org) was used to predict protein subcellular localization [26]. The pI and molecular weight were estimated using the Compute pI/Mw tool from ExPASy (http://web.expasy.org/compute_pi).

### Sequence alignments and phylogenetic analyses

Multiple alignment of ALDH protein sequences from *P*. *trichocarpa* and *A*. *thaliana* were performed using the Clustal X2.1 program [27]. The phylogenetic trees were constructed using the neighbor-joining method [28] in the MEGA package V5.2 [29] with bootstrap values from 1,000 replicate indicated at each node. The full length sequences of ALDH proteins used for phylogenetic analysis were listed in S1 Table.

### Bioinformatics analysis of *Populus ALDH* genes

The exon and intron structures were illustrated using Gene Structure Display Server (GSDS, http://gsds.cbi.pku.edu.cn) [30] by aligning the cDNA sequences with the corresponding genomic DNA sequences from Phytozome (http://phytozome.jgi.doe.gov/pz/portal.html#). The chromosomal locations of the *ALDH* genes were determined using the *Populus* genome browser (http://phytozome.jgi.doe.gov/pz/portal.html#!info?alias=Org_Ptrichocarpa). Tandem duplicated *PtALDH* genes were defined as adjacent homologous *ALDH* genes on the *Populus* chromosomes, with no more than one intervening gene. For synteny analysis, synteny blocks within the *Populus* genome and between *Populus* and *Arabidopsis* genomes were downloaded from the Plant Genome Duplication Database (PGDD, http://chibba.agtec.uga.edu/duplication/) [31] and those containing *Populus ALDH* genes were identified. The chromosomal locations of *ALDH* genes were drawn using Circos software [32]. To analyze the putative *cis*-acting regulatory elements, -1,000 nt of the upstream to +200 nt of the downstream of transcription start site (TSS) were searched using PlantCARE database [25].

### Publicly available microarray data analyses

The microarray data for various tissues and developmental stages available at NCBI Gene Expression Omnibus (GEO) database [33] under the series accession numbers GSE13990 and GSE13043 were used for the tissue-specific expression analysis. The series GSE13990 includes Affymetrix microarray data from nine different tissue samples representing three biological replicates [34], whereas series GSE13043 contains NimbleGen microarray data from five stem internodes (IN) from the apical bud to the base of the shoot (IN2 to IN5, IN9) in two biological replicates [35]. For abiotic and hormonal treatments, Affymetrix microarray data available in the NCBI GEO database under the series accession numbers GSE13109 (hypoxia), GSE17225 (drought), GSE26199 (heat) and GSE16786 were analyzed [36, 37]. GSE16786 is composed of the following five subsets: GSE14893 (nitrogen limitation, genotype 1979), GSE14515 (nitrogen limitation, genotype 3200), GSE16783 (1 week after leaf wounding), GSE16785 (90 h after leaf wounding), and GSE16773 (methyl jasmonate-elicited suspension cell cultures). Probe sets corresponding to *Populus ALDH* genes were identified using the online Probe Match tool POParray (http://aspendb.uga.edu/poparray). The probe sets corresponding to *Populus ALDH* genes were listed in S2 Table.

In Affymetrix GeneChip array, oligonucleotides of length 25 bp are used to probe genes. Typically, a gene is represented by a probe set composed of 11–20 probe pairs of these oligonucleotides [38]. To check the specificity of *PtALDHs* probe sets, we compared the recognition sites of *PtALDHs* probe sets between the paralogous genes. Except the probe sets corresponding to *PtALDH2B4* have four nonspecific binding sites in *PtALDH2B6*, all the other probe sets are highly specific to their corresponding genes (S1 Fig). So these probe sets could be used to reflect the real expression pattern.

### Plant material, RNA isolation and Real-time qRT-PCR

1-year-old *P*. *trichocarpa* grown in a growth chamber under long-day conditions (16 h light/8 h dark) at 23–25°C. Plant materials for qRT-PCR in different tissues (YL—young leaf, ML—mature leaf, PS—primary stem, SS—secondary stem, and R—root) were collected from 84K. Samples were frozen immediately in liquid nitrogen, and stored at -80°C for further analysis. Three biological replicates were performed.

Total RNA was extracted using the RNeasy Plant Mini Kit (Qiagen) with on-column treatment with RNase-free DNase I (Qiagen) to remove any contamination of genomic DNA. First-strand cDNA synthesis was carried out with approximately 1 μg RNA using the SuperScript III reverse transcription kit (Invitrogen) and random primers according to the manufacturer’s procedure. Primers with melting temperatures of 58–60°C and amplicon lengths of 100–250 bp were designed using Primer3 software (http://frodo.wi.mit.edu/primer3/input.htm). All primer sequences are listed in S3 Table.

Real-time qRT-PCR was conducted on 7500 Real Time PCR System (Applied Biosystems, CA, USA) using SYBR Premix Ex Taq Kit (TaKaRa, Dalian, China) according to the manufacturer’s instructions. Reactions were prepared in a total volume of 20 μl containing: 10 μl of 2×SYBR Premix, 2 μl of cDNA template, 0.4 μl of each specific primer to a final concentration of 200 nM. The reactions were performed as the following conditions: initial denaturation step of 95°C for 30 s followed by two-step thermal cycling profile of denaturation at 95°C for 10 s, and combined primer annealing/extension at 60°C for 34 s for 40 cycles. To verify the specificity of each primer pair, a melting curve analysis was performed ranging from 60°C to 95°C with temperature increasing steps of 0.06°C/s (5 acquisitions per °C) at the end of each run. The final threshold cycle (Ct) values were the mean of eight values including two biological replicates for each treatment and four technical replicates. The *PtActin* and *PtTubulin* gene were used as internal controls.

### Statistical analysis

The statistical significance of differences in measured parameters was tested by using the procedures of DPS (Zhejiang University, China). Differences between the means among different tissues or gene pairs were compared using Duncan test and Fisher’s protected least significant difference (LSD) test at 0.05 probability levels.

## Results and Discussion

### Characteristics of *ALDH* gene families in *Populus*

**Fig 1:**
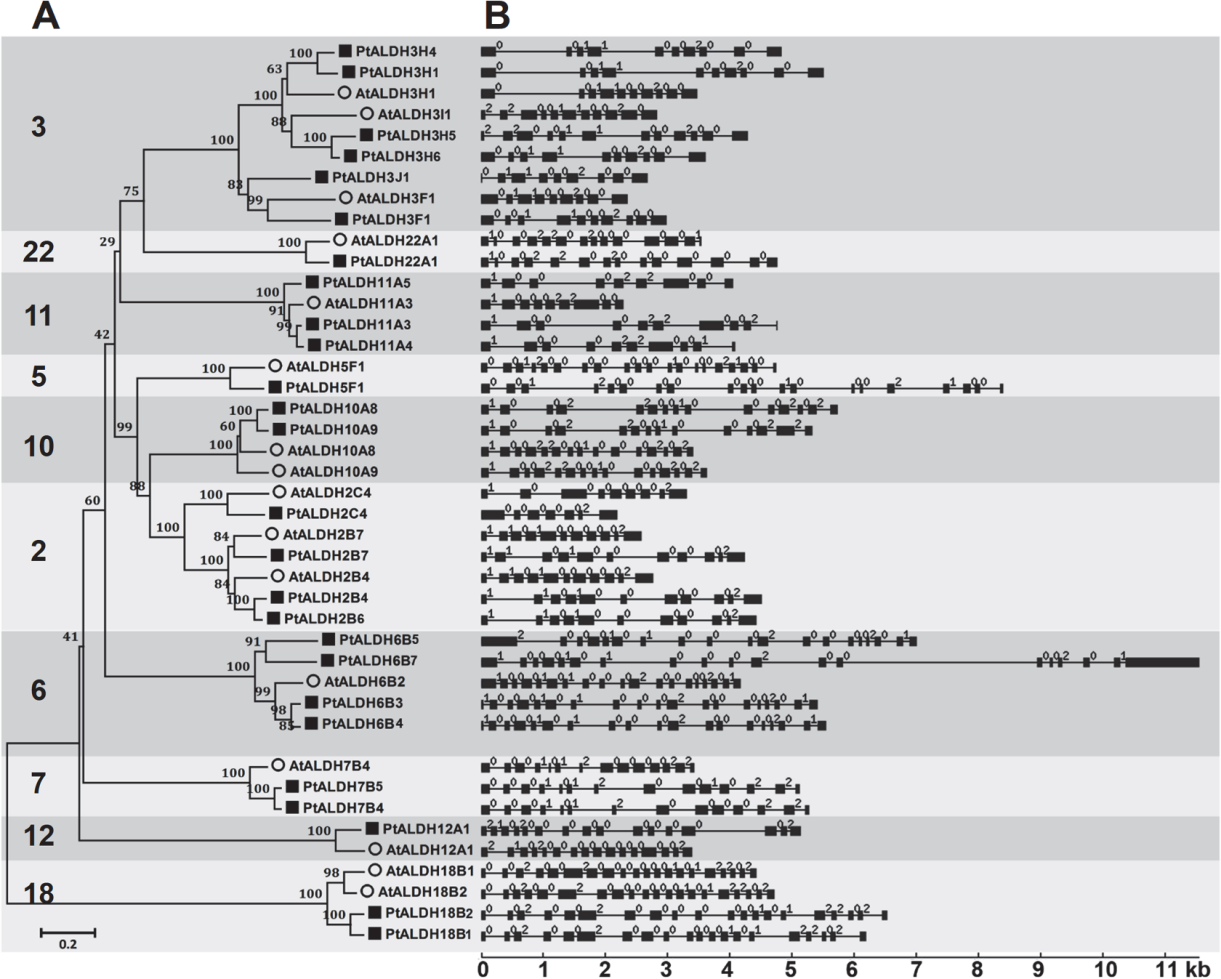
Phylogenetic analysis (A) and exon-intron structures (B) of *Arabidopsis* and *Populus ALDH* genes. **(A)** Multiple alignment of ALDH proteins from A. thaliana and P. trichocarpa was performed using Clustal X2.1. Phylogenetic tree was constructed using full-length protein sequences by the neighbor-joining (NJ) method with 1,000 bootstrap replicates using MEGA 5.2. Numbers above branches of the tree indicate bootstrap values. **(B)** Exon-intron structures of the *ALDH* genes. Only coding exons, represented by black boxes were drawn to scale. Lines connecting two exons represent introns. The numbers indicate the splicing phases of the *ALDHs*: 0, phase 0; 1, phase 1; and 2, phase 2.

Previous study identified 26 *ALDHs* in *P*. *trichocarpa* [25]. The *Populus ALDH* superfamily is larger than the *Arabidopsis ALDH* superfamily, the number of *ALDHs* in the *P*. *trichocarpa* genome was found to be consistent with 1.4~1.6 putative *Populus* homologs for each *Arabidopsis* gene according to previous comparative genomics studies [23]. The *ALDHs* identified in *P*. *trichocarpa* encode proteins that range from 401 to 958 amino acids (aa) in length, with predicted isoelectric points (pIs) from 5.44 to 9.12 (Table 1). In this study, we named the 26 *Populus ALDH* genes according to Brocker et al. [25]. The ALDH proteins from *Populus* were grouped into 10 families based on their protein sequence identities and phylogenetic relationships with *Arabidopsis* ALDHs (Fig 1 and S4 Table). Seven of the 10 *ALDH* families in *Populus* were represented by more than one gene (*ALDH3*, six genes; *ALDH2*, four genes; *ALDH6*, four genes; *ALDH11*, three genes; *ALDH7*, *ALDH10*, and *ALDH18*, two genes), whereas the remaining three families (*ALDH5*, *ALDH12*, and *ALDH22*) each were encoded by a single-copy gene.

**Table 1:** Populus ALDH genes and superfamilies.

Family	Gene Name	Gene Locus	CDS (bp)	ORF (aa)	pI	MW (kDa)	SubLoc*
Family 2	*PtALDH2B4*	Potri.012G078700.1	1611	536	8.74	58.65	mito: 8.5, chlo_mito: 7.5, chlo: 5.5
	*PtALDH2B6*	Potri.015G074100.1	1575	524	8.68	56.89	mito: 9.5, chlo_mito: 7.5, chlo: 4.5
	*PtALDH2B7*	Potri.002G189900.1	1623	540	6.11	58.84	mito: 9.5, chlo_mito: 6.5, chlo: 2.5
	*PtALDH2C4*	Potri.018G075000.1	1245	414	6.76	45.10	cysk: 8.0, cyto: 5.0
Family 3	*PtALDH3F1*	Potri.007G017100.1	1443	480	6.97	53.62	cyto: 5.0, golg: 5.0, plas: 2.0, chlo: 1.0
	*PtALDH3H1*	Potri.005G179300.1	1467	488	7.08	53.50	cyto: 9.0, chlo: 2.0, cysk: 2.0
	*PtALDH3H4*	Potri.002G081800.1	1467	488	7.08	53.97	cyto: 8.0, cysk: 3.0, chlo: 2.0
	*PtALDH3H5*	Potri.001G412900.1	1647	548	8.19	60.68	nucl: 6.0, chlo: 4.0, cyto: 3.0
	*PtALDH3H6*	Potri.005G069800.1	1467	488	5.68	54.12	cysk: 8.0, cyto: 5.0
	*PtALDH3J1*	Potri.001G259100.1	1206	401	9.12	44.21	chlo: 8.0, cyto: 3.0, extr: 2.0
Family 5	*PtALDH5F1*	Potri.010G174000.1	1611	536	8.46	57.50	mito: 10.0, chlo: 4.0
Family 6	*PtALDH6B3*	Potri.009G078600.1	1629	542	6.68	58.01	mito: 10.5, chlo_mito: 7.5, chlo: 3.5
	*PtALDH6B4*	Potri.001G283100.1	1629	542	7.00	57.90	mito: 12.0, chlo: 2.0
	*PtALDH6B5*	Potri.009G078700.1	2142	713	8.36	77.64	nucl: 7.0, chlo: 2.0, cyto: 2.0, cysk: 2.0
	*PtALDH6B7*	Potri.005G147700.1	2877	958	6.76	104.53	nucl: 11.0, chlo: 1.0, vacu: 1.0
Family 7	*PtALDH7B4*	Potri.003G067700.1	1527	508	5.71	54.69	nucl: 7.0, cyto: 3.0, chlo: 1.0, plas: 1.0, pero: 1.0
	*PtALDH7B5*	Potri.001G167100.1	1527	508	5.55	54.89	nucl: 7.0, chlo: 3.0, cyto: 2.0, plas: 2.0
Family 10	*PtALDH10A8*	Potri.012G075600.1	1512	503	5.46	54.93	chlo: 8.0, cyto: 4.0, pero: 2.0
	*PtALDH10A9*	Potri.015G070600.1	1620	539	5.44	59.13	chlo: 7.0, cyto: 4.0, pero: 2.0
Family 11	*PtALDH11A3*	Potri.018G109700.1	1521	506	7.86	54.30	cysk: 8.0, cyto: 5.0
	*PtALDH11A4*	Potri.006G186800.1	1497	498	7.84	53.64	cysk: 10.0, cyto: 4.0
	*PtALDH11A5*	Potri.018G020600.1	1497	498	6.72	53.44	cysk: 9.0, cyto: 5.0
Family 12	*PtALDH12A1*	Potri.015G064200.1	1704	567	7.65	62.97	mito: 8.5, chlo: 5.0, cyto_mito: 5.0
Family 18	*PtALDH18B1*	Potri.010G198400.1	2160	719	6.46	77.78	E.R.: 5.0, chlo: 3.0, nucl: 1.0, cyto: 1.0, mito: 1.0, plas: 1.0, extr: 1.0
	*PtALDH18B2*	Potri.008G060200.1	2148	715	6.05	77.46	chlo: 4.0, E.R.: 4.0, nucl: 2.0, cyto: 1.0, plas: 1.0, extr: 1.0
Family 22	*PtALDH22A1*	Potri.008G106000.1	1785	594	6.84	65.70	chlo: 4.0, cyto: 3.5, cyto_nucl: 2.5, plas: 2.0, vacu: 2.0, mito: 1.0

Notes: Gene loci are obtained from the Phytozome website (http://www.phytozome.net).

* PSORT predictions: plas (plasma membrane), vacu (vacuolar membrane), cyto (cytosol), chlo (chloroplast), cysk (cytoskeleton), pero (peroxisome), nucl (nuclear), E.R. (endoplasmic reticulum), mito (mitochondrion), golg (golgi), extr (extracellular).

The numbers indicate the number of nearest neighbors to the query which localize to each site.

To investigate the evolutionary relationships of ALDH proteins from different organisms, we summarized numbers of gene family members for each individual *ALDH* family in *P*. *trichocarpa* and seven other plant species (*Arabidopsis thaliana* [8], *Vitis vinifera* [10], *Zea mays* [39], *Oryza sativa* [18], *Physcomitrella patens*, *Chlamydomonas reinhardtii*, and *Ostreococcus tauri* [40]), three mammals (*Homo sapiens*, *Mus musculus*, and *Rattus norvegicus*) and fungi [7] (S5 Table). Plant *ALDHs* are grouped into 13 families: *ALDH2*, *ALDH3*, *ALDH5*, *ALDH6*, *ALDH7*, *ALDH10*, *ALDH11*, *ALDH12*, *ALDH18*, *ALDH21*, *ALDH22*, *ALDH23*, and *ALDH24*. *Populus* and other vascular plants share 10 common core *ALDH* families (i.e. families 2, 3, 5, 6, 7, 10, 11, 12, 18, and 22), suggesting that these 10 families may have evolved before the divergence of monocots and eudicots. Moreover, eight of the 10 core families also are shared by terrestrial plants and algae (families 2, 3, 5, 6, 10, 11, and 12), suggesting that these families have ancient origins that predate the transition of aquatic plants onto land. Interestingly, it was reported previously that *ALDH19* was also unique to plants; however, no *Populus* homologue of *ALDH19* was detected in either this study or other studies of vascular plants. To date, the only known member of *ALDH19* family is a single gene from tomato, suggesting that *ALDH19* may have evolved specifically in this lineage [41].

It is worth noting that vascular plants such as *P*. *trichocarpa*, *Z*. *mays*, and *V*. *vinifera* have more *ALDHs* than animals and fungi. To date, *Populus ALDH* families are the most expanded with 26 genes compared with the *ALDH* families in other well characterized plants (24 *ALDHs* in *Z*. *mays*, 23 in *V*. *vinifera*, 21 in *O*. *sativa*, 16 in *A*. *thaliana*, 20 in *P*. *patens*, eight in *C*. *reinhardtii*, and six in *O*. *tauri*). Unlike animals, plants cannot move to avoid exposure to environmental stresses and, as a result, plants may require many stress-response proteins to protect them when exposed to abiotic and biotic stresses [10, 42]. Compared with other annual plant species, *Populus* species undergo secondary growth, seasonal variation, and are exposed frequently to various environmental stresses. The expanded *ALDH* families in *Populus* imply that the *ALDHs* may be involved in developmental processes or improving stress tolerance in woody species.

### Phylogenetic and gene structure analyses of *Populus ALDH* genes

To examine the phylogenetic relationships among the *Populus ALDH* genes and other plant species, we generated a phylogenetic tree by aligning the full-length ALDH protein sequences identified in *P*. *trichocarpa* and *A*. *thaliana* [8]. As shown in Fig 1A, the ALDHs from the same families tended to cluster together. Among these families, *ALDH18* was the most distantly related family in the phylogeny. This finding is consistent with previous research in rice, which indicated that two OsALDH18 proteins had the greatest degree of sequence divergence from the other *ALDH* families and did not contain the conserved ALDH active sites [18]. A likely reason for this observation is that members of the *ALDH18* family may be involved in a variety of biological processes, which require that a very diverse range of substrates can be recognized in a sequence- and/or structure-specific manner.

Furthermore, we analyzed the exon-intron organization in the 26 *Populus ALDHs* and 16 *Arabidopsis ALDHs* to investigate their structural diversity (Fig 1B). Exon-intron structural divergence within families plays a pivotal role in the evolution of multiple gene families. Generally, the positions of some spliceosomal introns were conserved in orthologous genes and, in many cases, conservation of the exon-intron organization in paralogous genes was high enough to reveal the evolutionary relationship between introns [43]. As shown in Fig 1B, most of the members in some of the families (2, 3, 7, 10, 11, and 18) had the same number of exons and nearly identical exon lengths. We also examined intron phases with respect to codons are found that the intron phases were remarkably well conserved among family members, whereas the intron arrangements and intron phases were distinct between families (Fig 1B). The high degree of sequence identity and similar exon-intron structures of *ALDHs* within each family suggested that *Populus ALDH* families may have undergone gene duplications throughout evolution, resulting in *ALDH* gene families that contain multiple copies of similar genes with functions that partially or completely overlap. Previous studies reported that *ALDHs* from rice, grape, and *Arabidopsis* had highly conserved exon-intron structures [10, 18]. Here, we compared the exon-intron structures of the *Populus* and *Arabidopsis ALDHs* and found that the gene structures were conserved not only within a species but also across these two species (Fig 1B). However, we also identified exons that had been gained or lost during the evolution of several of the *ALDHs*. One such example is the *ALDH3* gene family in which *PtALDH3H5* and *PtALDH3H6* seem to have acquired one additional exon at their 3’-end, while *PtALDH3H6* lost the first two exons and *PtALDH2C4* may have lost two exons at their 5’-ends.

### Chromosomal location and expansion patterns of *Populus ALDH* genes

**Fig 2:**
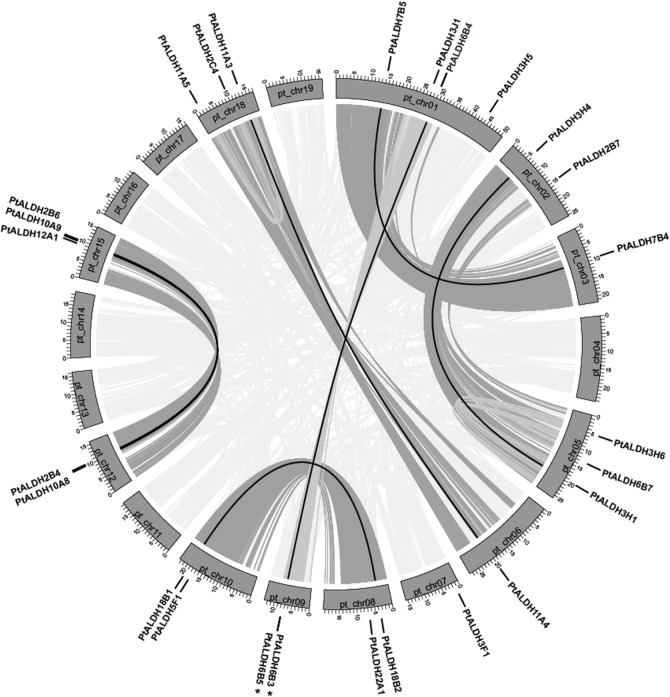
Distribution and synteny of *ALDH* genes on *Populus* chromosomes. Nineteen *Populus* chromosomes (chr01-chr19) are depicted as grey bars. Chromosome numbers are indicated in centers of each bar. *Populus ALDH* genes are indicated by vertical black lines outer the circles. Duplicate pairs formed by whole or segmental genome duplication are connected by black lines. Grey lines connected between chromosomes denote syntenic regions of the *Populus* genome.

*In silico* mapping of the gene loci showed that the 26 *Populus ALDHs* were mapped unevenly to 12 of 19 *Populus* chromosomes (chr). Chr1 had the largest number of four *ALDHs* followed by three *ALDHs* on chr5, chr15, and chr18. In contrast, only one *ALDH* mapped to chr3, chr6, and chr7 and two *ALDHs* mapped to chr2, chr8, chr9, chr10, and chr12 (Fig 2). No substantial clustering of the *Populus ALDH* genes was observed, even on the chromosomes with high densities of *ALDHs*.

Previous analysis indicated that the *Populus* genome may have undergone at least three rounds of genome-wide duplications followed by multiple segmental duplication, tandem duplication, and transposition events such as retroposition and replicative transposition [44]. To determine the evolutionary relationships among the *ALDHs*, we mapped *Populus ALDHs* to the duplicated blocks reported previously [23]. The distributions of the *ALDHs* relative on the corresponding duplicated blocks are shown in Fig 2. Within the duplicated blocks that were reported to be associated with recent salicoid duplication events, 92.3% (24 of 26) of the *Populus ALDHs* were preferentially retained duplicate genes that were located in both duplicated regions; only two *ALDHs* (*PtALDH3F1* and *3H5*) were located outside any of the duplicated blocks. Eight duplicated blocks contained *ALDHs* (*PtALDH2B7*, *2C4*, *3J1*, *3H6*, *5F1*, *6B7*, *12A1*, and *22A1*) on only one of the blocks and lacked duplicate genes on the corresponding blocks. These results indicated that dynamic rearrangement may have occurred following the segmental duplication and this resulted in the loss of some genes.

Two tandem *ALDH* gene duplications have been reported in rice (*OsALDH2-1*/*2-2* and *OsALDH3-1*/*3-2*) [18] and grape (*VvALDH5F1*/*5F2*/*5F3* and *VvALDH6B3*/*6B5*) [10]. In the present study, we also identified tandem duplications in the *Populus ALDH6* gene family (*PtALDH6B3*/*6B5*) (Fig 2 and Table 2). Analysis of *ALDH* paralogous pairs showed that seven of 10 gene pairs wer located in conserved positions on segmental duplicated blocks, indicating that these genes might have been generated by genome duplication (Fig 2 and Table 2). The high retention rate (14/26, 53.8%) of the duplicated *ALDHs* was consistent with recent reports of other gene families in *Populus* [45–47]. In summary, the seven *Populus* multi-member *ALDH* families (Table 2) all were associated with either segmental or tandem duplication events, indicating that segmental and tandem duplications may have played important roles in the expansion of *ALDHs* in *Populus*.

**Table 2:** Divergence between paralogous *ALDH* genes pairs in *Populus*.

Family	Gene 1	Gene 2	Duplication	*K*a	*K*s	*K*a/*K*s	Date (million years ago)
2	*PtALDH2B4*	*PtALDH2B6*	W	0.047	0.281	0.169	15.44
3	*PtALDH3H1*	*PtALDH3H4*	W	0.065	0.206	0.316	11.34
3	*PtALDH3H5*	*PtALDH3H6*	O	0.053	0.224	0.235	12.32
6	*PtALDH6B3*	*PtALDH6B4*	W	0.033	0.218	0.151	11.99
6	*PtALDH6B5*	*PtALDH6B7*	O	0.189	1.627	0.116	89.41
6	*PtALDH6B3*	*PtALDH6B5*	T	0.162	1.441	0.113	79.15
7	*PtALDH7B4*	*PtALDH7B5*	W	0.020	0.226	0.088	12.40
10	*PtALDH10A8*	*PtALDH10A9*	W	0.034	0.153	0.221	8.38
11	*PtALDH11A3*	*PtALDH11A4*	W	0.018	0.188	0.096	10.35
18	*PtALDH18B1*	*PtALDH18B2*	W	0.042	0.270	0.154	14.85

Notes: Gene pairs were identified at the terminal nodes of the phylogenetic tree shown in Fig 1. Synonymous (*K*s) and nonsynonymous substitution (*K*a) rates are presented for each pair. Gene pairs created by tandem duplication (T), whole genome duplication (W), or other (O) events are indicated in the table.

### Duplication and evolution analysis of the *Populus ALDH* genes

Duplicated genes may undergo divergent fates such as nonfunctionalization (loss of original functions), neofunctionalization (acquisition of novel functions), or subfunctionalization (partition of original functions) [48, 49]. To determine whether positive selection was involved in the divergence of *ALDHs* after duplication, the nonsynonymous (*K*a) to synonymous (*K*s) ratios were calculated for paralogous *PtALDH* gene pairs [50]. *K*a/*K*s = 1 indicates neutral selection, *K*a/*K*s >1 indicates accelerated evolution with positive selection, and *K*a/*K*s <1 indicates purifying selection [51]. The *K*a/*K*s ratios of all 10 *PtALDH* gene pairs were less than 1 (Table 2), implying that *Populus ALDH* gene pairs may have evolved mainly under the influence of purifying selection.

Based on the divergence rate of 9.1×10^–9^ synonymous mutations per synonymous site per year proposed previously for *Populus* [52], we estimated the evolutionary dates of the segmental duplication events using *K*s as the proxy for time (Table 2). We found that seven of the 10 paralogous pairs (*PtALDH2B4*/*2B6*, *PtALDH3H1*/*3H4*, *PtALDH3H51*/*3H6*, *PtALDH6B3*/*6B4*, *PtALDH7B4*/*7B5*, *PtALDH11A3*/*11A4*, *PtALDH18B1*/*18B2*) had very consistent *K*s values (from 0.188 to 0.281), suggesting that these duplication events occurred in *Populus* within the last 10.35 to 15.44 million years. This period is consistent with the time (13 million years) when a recent large-scale genome duplication event is thought to have occurred in *Populus* [53].

### Evolutionary relationship between the *Populus* and *Arabidopsis ALDH* gene families

**Fig 3:**
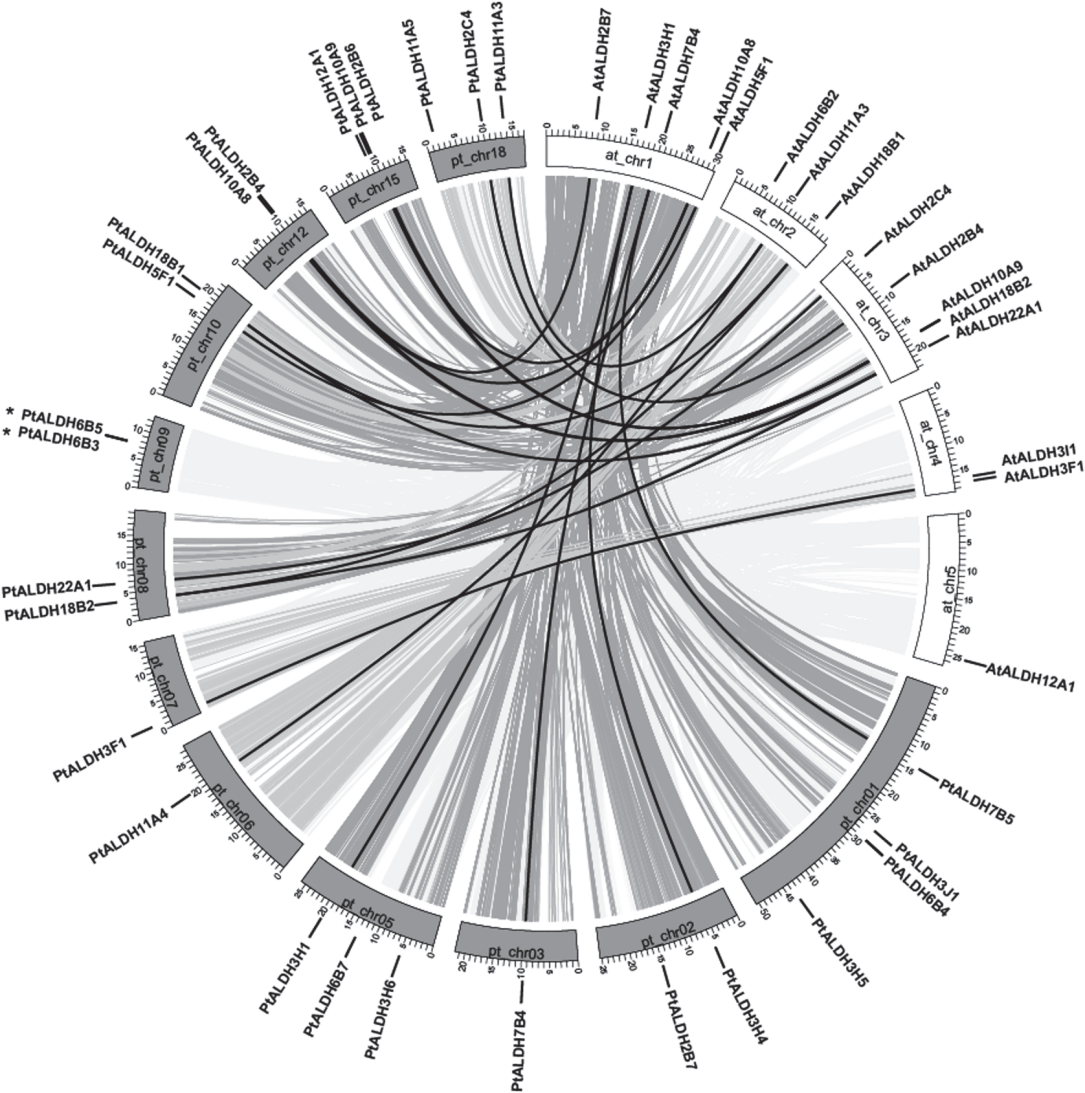
Synteny analysis of *ALDH* genes between *Populus* and *Arabidopsis*. *Populus* and *Arabidopsis* chromosomes are depicted as grey and blank boxes, respectively. *ALDH* genes are indicated by vertical black lines outer the circles. Grey lines connected between *Populus* and *Arabidopsis* chromosomes denoted syntenic relationships.

By comparing the genome sequences from different taxa it is possible to reconstruct the evolutionary history of each gene in its entirety [54]. To further investigate the origin and evolution of *Populus ALDHs*, we analyzed a comparative synteny map of the *Populus* and *Arabidopsis* genomes (Fig 3). *Arabidopsis* is an important model plant species and the functions of most *Arabidopsis ALDH* genes have been well characterized [8]. Thus, the comparative genomics analysis allowed us to infer the functions of the *Populus ALDHs* based on the annotations of their homologs in *Arabidopsis*.

Large-scale syntenies of orthologs from eight *ALDH* families (*ALDH2*, *3*, *5*, *7*, *10*, *11*, *18*, and *22*) in the *Arabidopsis* and *Populus* genomes were identified (Fig 3). The syntenies were unambiguous and included the following orthologous pairs: *AtALDH2C4*-*PtALDH2C4*, *AtALDH3F1*-*PtALDH3F1*, *AtALDH5F1*-*PtALDH5F1* and *AtALDH22A1*-*PtALDH22A1* (Fig 3), indicating that these genes/families were in the genome of the last common ancestor of *Populus* and *Arabidopsis*. Three of the *Populus ALDH* paralogous gene pairs were syntenic with *Arabidopsis ALDHs* (*AtALDH3H1*-*PtALDH3H1*/*3H4*, *AtALDH7B4*-*PtALDH7B4*/*7B5* and *AtALDH11A3*-*PtALDH11A3*/*11A4*), and these three *PtALDH* gene pairs were probably duplicated in the recent large-scale genome duplication event (13 million years ago) in *Populus* (Table 2). The syntenic interpretation was more challenging where duplicated *Populus* genes corresponded to two *Arabidopsis ALDHs* (e.g. *AtALDH10A8*/*10A9*-*PtALDH10A8*/*10A9*, *AtALDH18B1*/*18B2*-*PtALDH18B1*/*18B2*). The remaining two families (*ALDH6* and *ALDH12*) did not map to any of the synteny blocks. However, it was not possible to conclude that these two *ALDH* families from *Populus* and *Arabidopsis* did not share a common ancestor because, after speciation, the *Populus* and *Arabidopsis* genomes may have undergone multiple rounds of significant chromosomal rearrangement and fusions, followed by selective gene loss [23].

### *Populus ALDH* genes were differentially expressed in different tissues

Whole-genome microarray assays have been used successfully to study gene expression profiles in *Populus* [45, 47]. To gain insight into the expression patterns of *Populus ALDH* genes in different tissues, a comprehensive analysis was conducted based on an Affymetrix (GSE13990) and a Nimblegen (GSE13043) microarray data generated by Wilkins and Dharmawardhana [34, 35]. Although these two microarray datasets were generated on different platforms, they largely represent the *Populus ALDHs* presented in this study.

**Fig 4:**
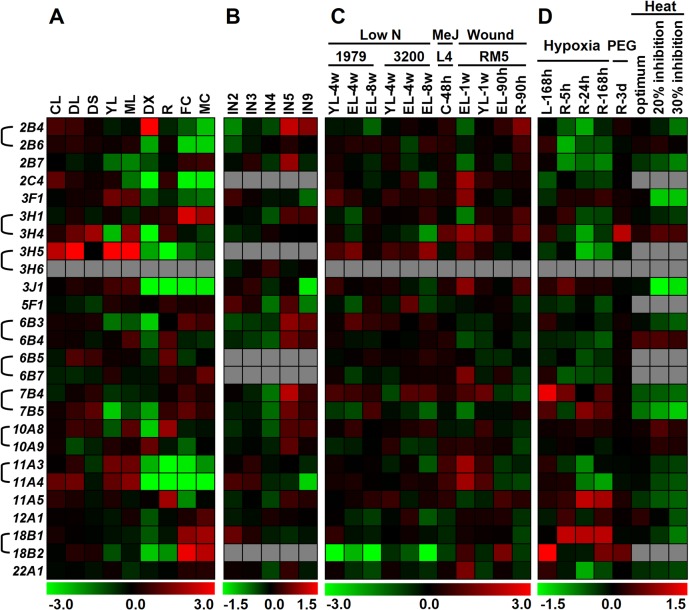
Expression profiles of *Populus ALDH* genes across different tissues and various abiotic stresses. **(A)** Heatmap showing expression profiles of *PtALDH* genes across various tissues analyzed. The Affymetrix microarray data were obtained from NCBI Gene Expression Omnibus (GEO) database under the series accession number GSE13990. CL, continuous light-grown seedling; DL, etiolated dark-grown seedling transferred to light for 3 h; DS, dark-grown seedlings; YL, young leaf; ML, mature leaf; DX, differentiating xylem; R, root; FC, female catkins; MC, male catkins. **(B)** Heatmap showing expression profiles of *PtALDH* genes at different stem development stages. The NimbleGen microarray data were obtained from NCBI GEO database under the series accession number GSE17230. IN2-IN9, stem internodes 2 to stem internodes 9. **(C)** Heatmap showing expression profiles of *PtALDH* genes across various stresses and genotypes analyzed. Microarray data under the series accession number GSE16786 was obtained from NCBI GEO database. Genotypes analyzed included: *P*. *fremontii* × *angustifolia* clones 1979, 3200, and RM5, *P*. *tremuloides* clones 271 and L4, and *P*. *deltoids* clones Soligo and Carpaccio. Tissues analyzed included: YL, young leaves; EL, expanding leaves; R, root tips; C, suspension cell cultures. Stress treatments included: low N, nitrogen limitation; MeJ, Methyl Jasmonate elicitation; Wound, sampled either one week or 90 hours after wounding. **(D)** Heatmap showing expression profiles of *PtALDH* genes under hypoxia, drought and heat stresses. Microarray data under the series accession number GSE13109 (hypoxia), GSE17225 (drought) and GSE26199 (heat) was obtained from NCBI GEO database. Stress treatments included: Hypoxia, the root system of grey poplar (*Populus* × *canescens*) were flooded for up to 168 h; Drought, a moderate water deficit was applied by adding PEG to the nutrient solution (200g/l) on hybrid poplar (*P*. *deltoides* × *P*. *nigra*) grown in hydroponics; Heat, fully expanded leaf samples of *P*. *trichocarpa* were harvested at 4 physiological states as determined from prior gas exchange measurements (growth temperature, 22°C—baseline, 31.75°C—photosynthetic optimum, 38.4°C—20% inhibition of optimum and 40.5°C—30% inhibition of optimum). Color scale represents log2 expression values, green represents low level and red indicates high level of transcript abundances.

Most *Populus ALDHs* show distinct tissue-specific expression patterns. As shown in Fig 4A, five genes (*PtALDH3F1*, *3H5*, *3J1*, *11A3* and *11A4*) showed relatively high expression levels in young and mature leaves but low expression level in differentiating xylem, root, and male and female catkins. Four genes (*PtALDH6B4*, *6B5*, *10A8*, and *11A5*) were expressed mainly in root, three genes (*PtALDH3H1*, *18B1*, and *18B2*) had relatively high expression levels in male and female catkins, and only *PtALDH2B4* and *10A9* exhibited high expression levels in differentiating xylem. The tissue-specific expression patterns for these genes implied their involvement in special developmental processes. Genes in single-member families (*PtALDH5F1*, *12A1*, and *22A1*, Fig 4A) tended to maintain consistent expression levels across diverse organs, probably because of functional constraints, suggesting that these genes may participate in the basic metabolism of *Populus*.

To identify putative *Populus ALDHs* involved in stem development, a heat map was generated based on the microarray data (GSE13043). As shown in Fig 4B, most of the *ALDHs* exhibited different expression levels in *Populus* stem segments (IN2-IN5, and IN9), where IN2 and IN3 represent the vascular tissues of primary growth, mainly primary xylem and primary phloem, and IN5 and IN9 represent well developed secondary phloem and secondary xylem vessels, as well as fibers with well lignified secondary cell walls [35]. The expression profiles of these *ALDHs* suggested they may play special roles during each stage of cell wall biosynthesis. Of the *Populus ALDHs* we examined in this study, 10 were predominantly expressed in IN5 (*PtALDH2B4*, *2B7*, *3H1*, *6B3*, *6B4*, *7B4*, *7B5*, *10A8*, *10A9*, and *11A5*), suggesting they may be involved in the transition from primary to secondary growth (Fig 4B). The expression patterns of nine *PtALDH* paralogous gene pairs in five tissues (YL—young leaf, ML—mature leaf, PS—primary stem, SS—secondary stem, and R—root) were also examined by qRT-PCR and the mRNA levels of the detected genes were generally consistent with the results from the microarray data. Nair et al. [55] reported that *AtALDH2C4* (*REF1*, At3g24503) was involved in the formation of both soluble and cell wall-linked ferulate esters. In maize, the ortholog of *Arabidopsis ALDH22A1* was highly expressed in caffeic acid *O*-methyltransferase deficient tissues, and was also the most expressed *ALDH* in normal internodes [56]. Similar to *AtALDH2C4* in *Arabidopsis*, the ortholog *ZmALDH2C2* (*RF2C*) in maize also was reported to be involved in the biosynthesis of ferulic acid, a major esterified hydroxycinnamic acid in cell walls that impedes the hydrolysis of the cell wall biomass [57].

### *Populus ALDH* genes potentially involved in response to abiotic stresses

Plant *ALDHs* have been reported to play important roles in the adaptation of plants to various abiotic stresses [8, 18]. Here, we analyzed the expression profiles of *PtALDHs* under abiotic stresses such as low nitrogen, methyl jasmonate (MeJ) treatment, mechanical wounding, hypoxia, drought, and heat (series accession numbers GSE13109, GSE17225, GSE26199 and GSE16786) [36, 37]. *PtALDH18B1* was commonly down-regulated under nitrogen deprivation stress in 4-week-old young leaves, 4-week and 8-week-old expanded leaves in two different *Populus* genotypes (1979 and 3200, Fig 4C). Other *PtALDHs* showed different response profiles to nitrogen deficit stress between these two *Populus* genotypes. For instance, *PtALDH3H5* and *7B4* were significantly up-regulated in 8-week-old expanded leaves in genotype 3200, whereas no distinctive expression patterns were observed in genotype 1979. In response to MeJ feeding in cell culture, three genes (*PtALDH3H4*, *11A3*, and *11A4*) were found to be up-regulated (Fig 4C). Mechanical wounding stress commonly caused up-regulation of 12 genes at 1 week after wounding in expanded leaves. In addition, five genes (*PtALDH2B4*, *2B6*, *3J1*, *3H1*, and *3H4*) were up-regulated at 90 hours after wounding in root tips (R), suggesting the functional divergence of *PtALDHs* in response to mechanical wounding.

In response to hypoxia, two genes (*PtALDH7B4* and *18B2*) were up-regulated significantly in leaves at 168 hours after hypoxia, while three other genes (*PtALDH7B5*, *11A5*, and *18B1*) were up-regulated during hypoxia in roots (Fig 4D). Drought stress caused up-regulation of two genes (*PtALDH3H4* and *18B2*) at 3 days after polyethylene glycol (PEG) in roots (Fig 4D). In a previous study, the physiological condition was divided into four states based on the *Populus* photosynthetic activity at temperatures from 22°C to 42°C: baseline (22°C, the growth temperature), optimum (31.75°C, temperature at which the maximum net CO_2_ assimilation rate is observed), 20% inhibition of optimum (38.4°C), and 30% inhibition of optimum (40.5°C) [37]. Most *PtALDHs* were down-regulated under heat stress and only two genes (*PtALDH3H4* and *6B4*) were up-regulated when photosynthesis was inhibited by 20% and 30% (Fig 4D). *ALDHs* that were induced under various stresses have been identified in many plant species, indicating that they may play critical roles in plant adaptation to these stresses [58]. In *Arabidopsis*, it was reported that overexpression of *ALDH3I1* may improve the plant’s tolerance to diverse stresses [13]. In addition, both *ALDH3* and *ALDH7* were found to be involved in stress-regulated detoxification pathways. In *Arabidopsis*, the chloroplastic *ALDH3I1* and the cytoplasmic *ALDH7B4* may confer tolerance to osmotic and oxidative stresses [14]. *ALDH18* gene encodes P5CS (Δ^1^-pyrroline-5-carboxylate synthetase), a key regulatory enzyme that plays a crucial role in proline biosynthesis. Recent studies indicated that *P5CS1* was required for proline biosynthesis under osmotic stress in *Arabidopsis*, suggesting *ALDH18* may also be abiotic stress responsive [59]. Our findings are largely consistent with studies in *Arabidopsis*, rice, and grape that indicated *ALDH* genes from families 2, 3, 6, 7, 11, and 18 were significantly induced in abiotic stressed plants [10, 14, 18, 58, 59]. Our expression data indicated that some *Populus ALDH* genes are potential candidates for improving *Populus* tolerance to abiotic stresses. Extensive further studies are also required to examine the exact biochemical roles of *Populus ALDHs* in developmental processes and stress tolerance.

### Divergent expression of *PtALDH* gene pairs

**Fig 5:**
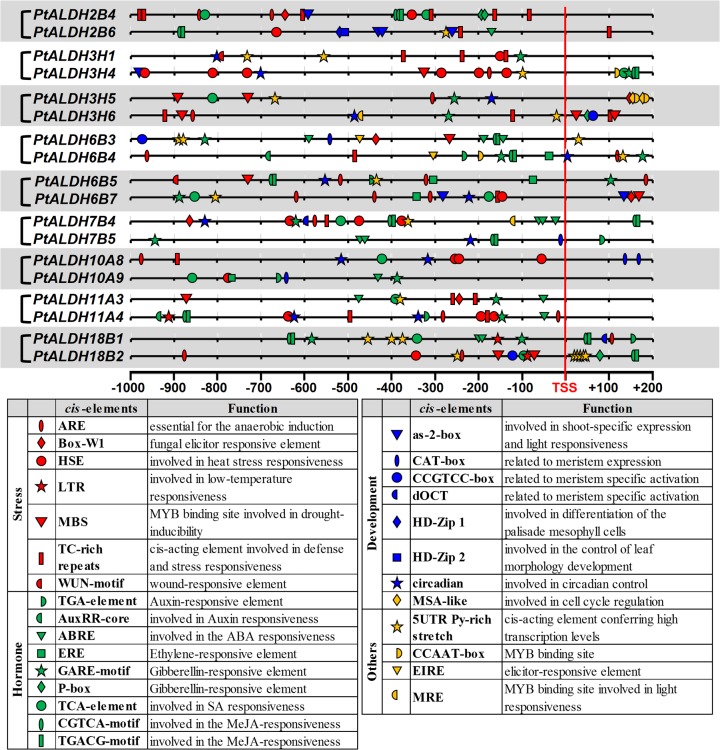
Comparisons of the *cis*-acting elements in the promoter regions of *PtALDH* gene pairs. Schematic representation of nine *PtALDH* gene pairs promoters. A selection of putative *cis*-acting elements identified in the PlantCARE database is indicated. Distances shown in the figure are relative to the Transcription Start Site (TSS).

**Fig 6:**
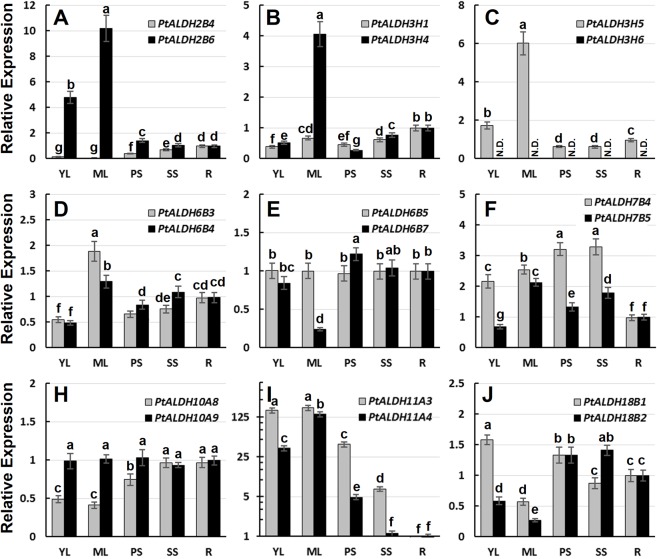
Expression analysis of nine *PtALDH* gene pairs in different tissues using qRT-PCR. The relative mRNA abundance of nine *PtALDH* gene pairs were quantified in vegetative tissues (YL, young leaves; ML, mature leaves; PS, primary stem; SS, secondary stem; R, roots). The average expression of each gene was calculated relatively to the first biological replicate of roots ± standard error (SE) (n≥3). Relative expression represents log2 expression values. Bars with the same letters are not significantly different according to Duncan test and Fisher’s protected LSD test (*P*<0.05).

Previous studies of some closely-related *ALDHs* hinted at potential roles of functional specialization in the retention of duplicated genes [11, 60]. Most *Populus ALDHs* arose from recent genome duplication and tandem duplication events (Table 2). Duplicated *ALDHs* showed different tissue-specific expression patterns (Fig 4), suggesting that gene duplications supplied opportunities for the duplicates to be free from the functional constraints of the parent gene. To analyze the expression divergence between *PtALDH* paralogous gene pairs, we identified the putative *cis*-acting elements in the promoter regions of nine *PtALDH* gene pairs (from -1,000 nt upstream to +200 nt downstream of the transcription start site) using PlantCARE database (Fig 5). The nine *PtALDH* gene pairs all had different *cis-acting* elements in their promoter regions. We also examined the mRNA levels of the *PtALDH* gene pairs in different tissues by qRT-PCR to validate their expression patterns (Fig 6). For the *PtALDH2B4*/*PtALDH2B6* pair, several leaf development-related *cis*-acting elements (three as-2-boxes: involved in shoot-specific expression and light responsiveness, one HD-Zip1: involved in differentiation of the palis and mesophyll cells, and one HD-Zip2: involved in the control of leaf morphology development) were detected in the promoter of *PtALDH2B6* (Fig 5) but not in the *PtALDH2B4* promoter. As expected based on this finding, *PtALDH2B6* was highly expressed in young and mature leaves (Fig 6A), whereas *PtALDH2B4* had low mRNA levels because it lacked leaf development-related *cis*-acting element in its promoter. For the *PtALDH3H1*/*PtALDH3H4* pair, six HSE (involved in heat stress responsiveness) and one MBS (MYB binding site involved in drought-inducibility) were detected in the promoter of *PtALDH3H4* (Fig 5) but not in the promoter of *PtALDH3H1*; therefore, *PtALDH3H4* was significantly induced by heat and drought (PEG treatment) stresses, whereas *PtALDH3H1* was not (Fig 4D). In addition, many hormone related *cis*-acting elements, development related *cis*-acting elements, and stress response *cis*-acting elements were detected in the promoters of various *PtALDHs*, implying that different members of the *Populus ALDH* families were involved in different development processes and stress responses. Based on these data, we propose that expression divergence and/or functional specialization may have played important roles in the retention of the *Populus ALDH* duplicate genes. However, the functions of the *PtALDH* genes associated with development and stress responses in *Populus* need to be investigated further.

Functional diversification among gene family members is considered an important source of evolutionary innovation in complex organisms, and various theoretical models have been proposed to explain the mechanisms involved [61–64]. The most plausible models proposed for the retention of duplicated genes invoke sub- or neo-functionalization [24]. In this study, clear divergence in expression patterns was observed among the *Populus ALDHs* in different tissues and in response to different stress treatments. These findings clearly support the assertion that expression divergence is often the first step in functional divergence between duplicate genes and that this divergence increases the chance of duplicate genes being retained in a genome [61]. Our findings provide evidence for the evolutionary partitioning of ancestral functions among duplicated genes.

## Conclusions

Aldehyde dehydrogenases (*ALDHs*) are members of the NAD(P)^+^-dependent protein superfamily that catalyze the oxidation of a wide range of endogenous and exogenous highly reactive aliphatic and aromatic aldehyde molecules. Although the *ALDH* gene superfamily has been reviewed in many plants, no systematic analyses have been conducted to date in *Populus*, a model tree. In the present study, comprehensive analyses including phylogeny, gene structure, chromosomal location, expression profiles, and the *cis*-acting elements of members of the *Populus ALDH* gene superfamily were performed. A total of 26 *Populus ALDH* genes were grouped into 10 families. We found that the exon-intron structures were relatively conserved within each family. Comparative analysis showed that 10 paralogous gene pairs were created by different duplication types. An additional comprehensive analysis of the expression profiles provided insights into the possible functional divergence among members of the *ALDH* gene superfamily. Gene specific promoter *cis*-acting elements may explain the divergent expression patterns observed between eight of nine *PtALDH* gene pairs (one pair may have been generated by tandem duplication). Although the functions of the *PtALDHs* remain largely unknown and many experiments are needed to determine their exact functions, our phylogenetic and expression analyses may help in the selection of appropriate candidate genes for further functional characterization.

## Supporting Information

S1 Fig The specificity of probe sets between gene pairs. (PDF)

S1 Table List of ALDH protein sequences identified from *P*. *trichocarpa* and *A*. *thaliana*. (XLSX)

S2 Table Probe sets corresponding to *Populus ALDH* genes. (XLSX)

S3 Table The sequences of qRT-PCR primers. (DOCX)

S4 Table Amino acid identity among *P*. *trichocarpa* and *A*. *thaliana* ALDH proteins was analyzed in pairwise fashion. (XLSX)

S5 Table Number of *ALDH* family members identified in various organisms. (DOCX)
