# Supplementary material for: Aldehyde Dehydrogenase Gene Superfamily in Populus: Organization and Expression Divergence between Paralogous Gene Pairs
Source: PLoS One. 2015 Apr 24;10(4):e0124669. doi: 10.1371/journal.pone.0124669 (PMC4409362; doi:10.1371/journal.pone.0124669)

Probe sets of *PtALDH2B4:*

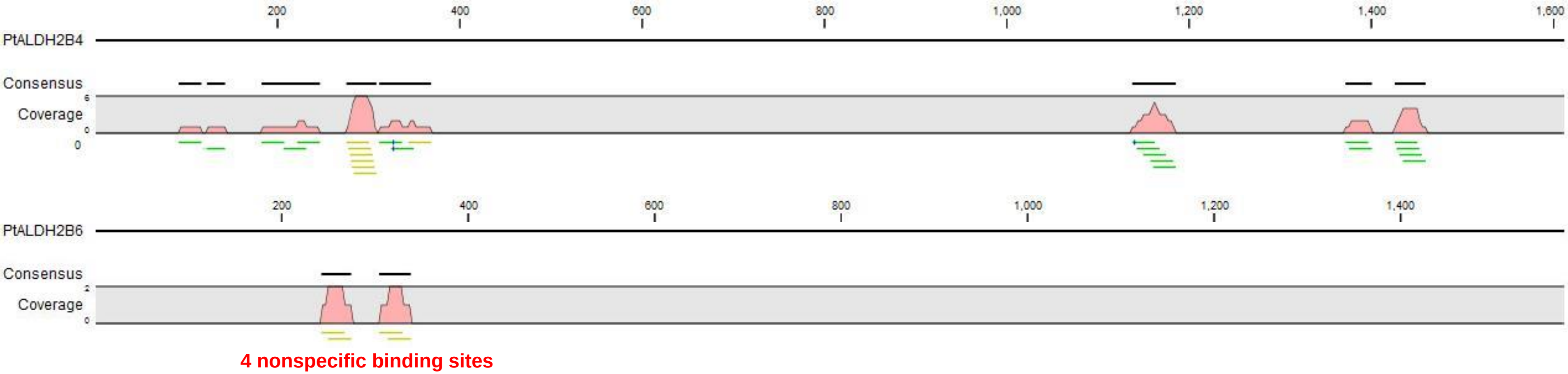

Probe sets of *PtALDH2B6:*

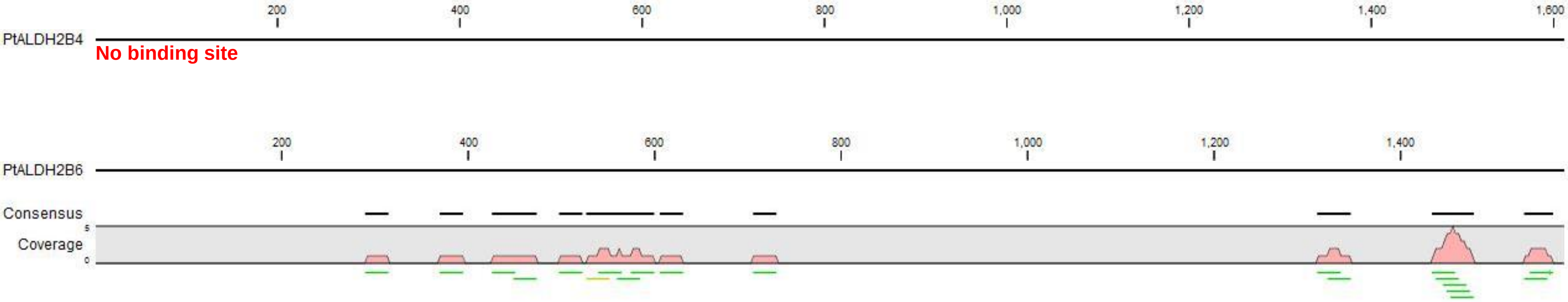

Probe sets of *PtALDH3H1:*

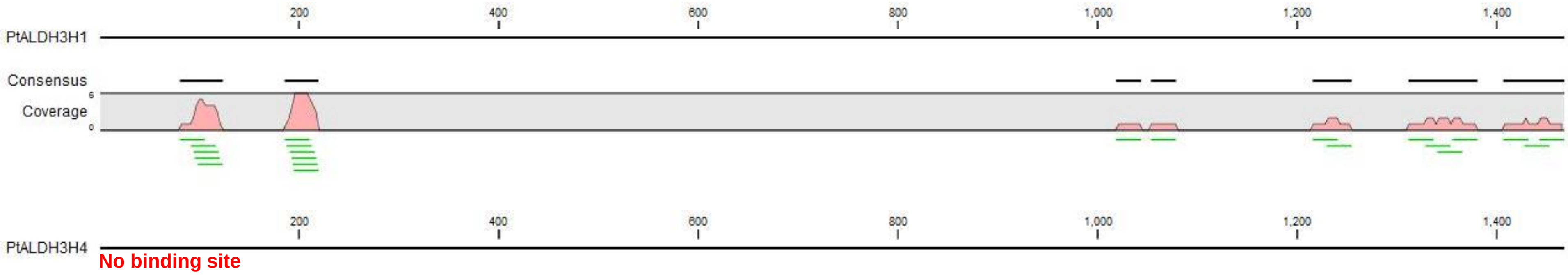

Probe sets of *PtALDH3H4:*

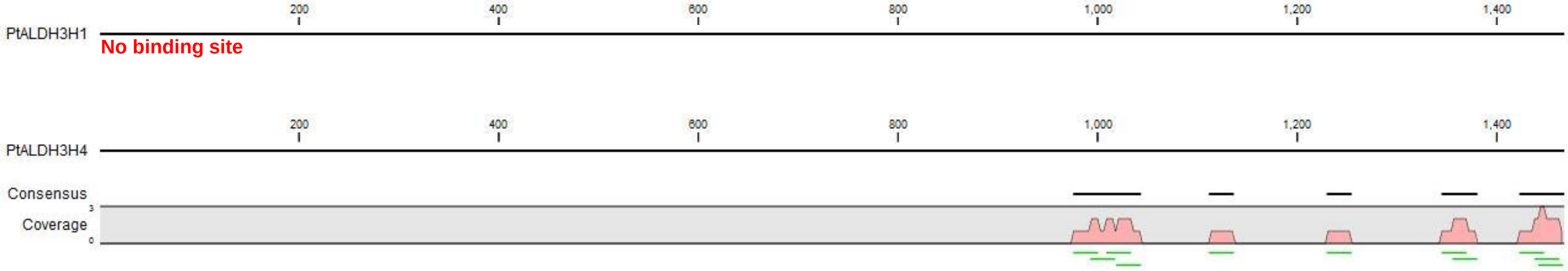

Probe sets of *PtALDH3H5:*

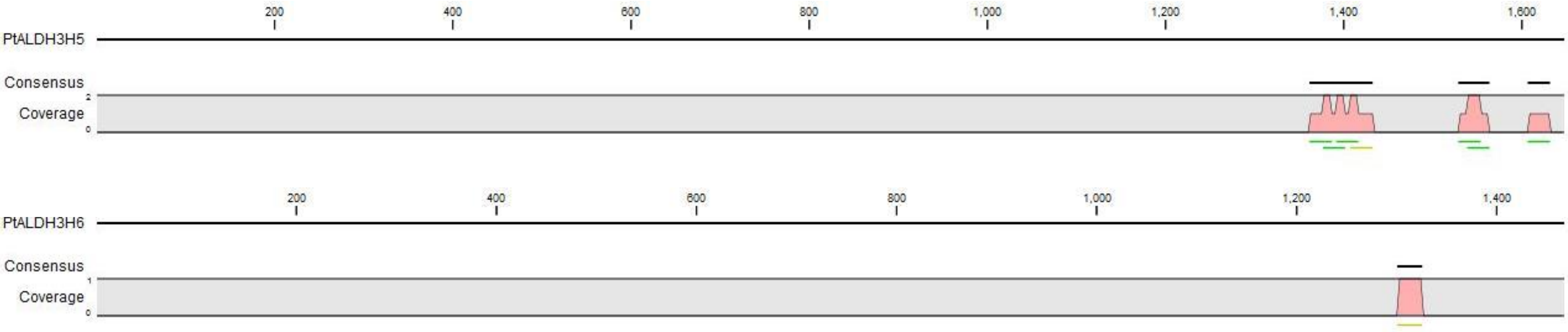

1 nonspecific binding sites

Probe sets of *PtALDH3H6:*

No corresponding probe sets

Probe sets of *PtALDH6B3:*

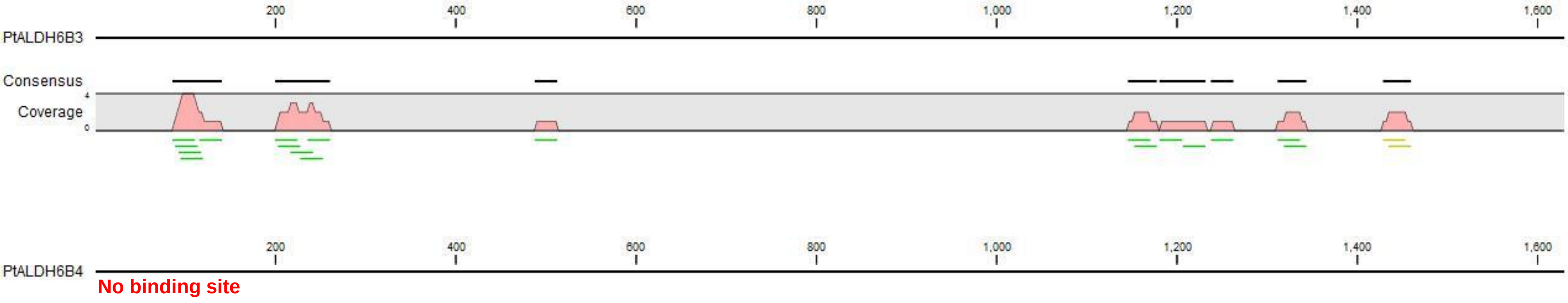

Probe sets of *PtALDH6B4:*

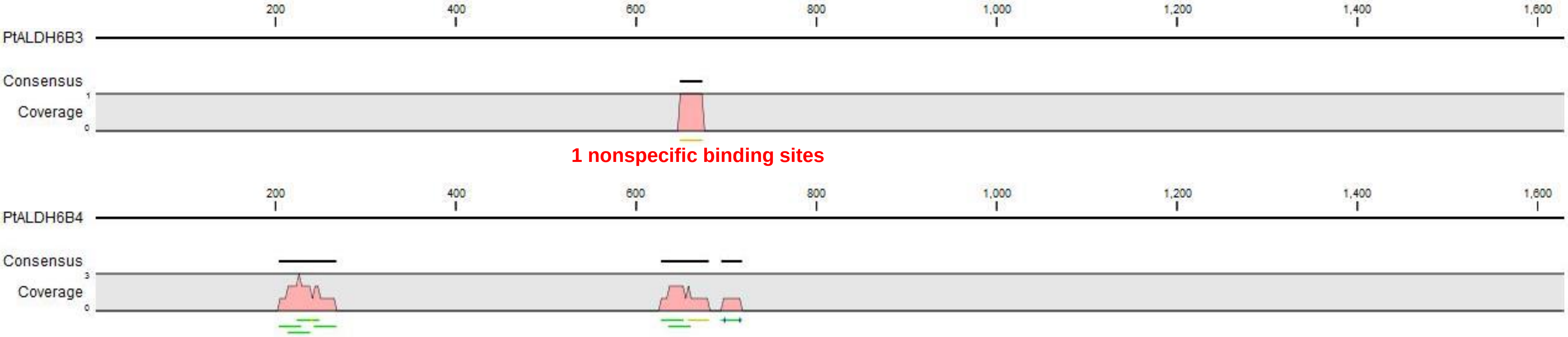

Probe sets of *PtALDH6B5:*

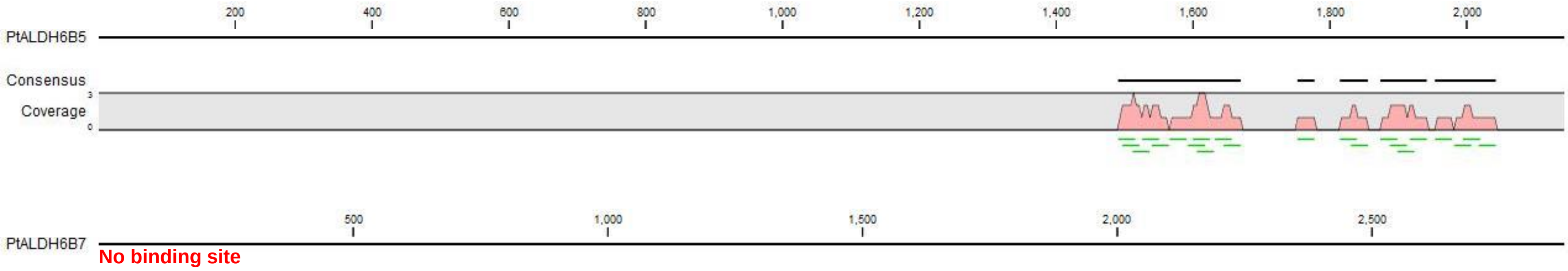

Probe sets of *PtALDH6B7:*

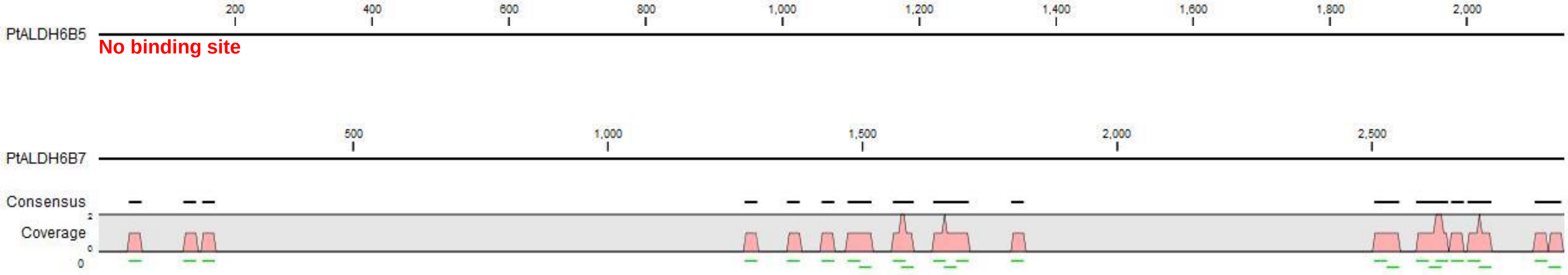

Probe sets of *PtALDH10A8:*

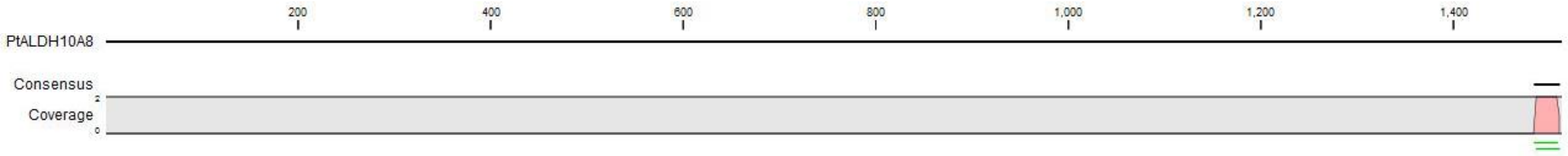

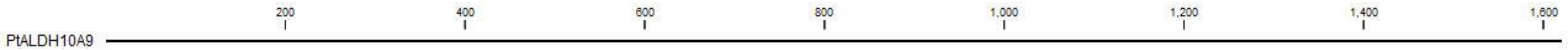

No binding site

Probe sets of *PtALDH10A9:*

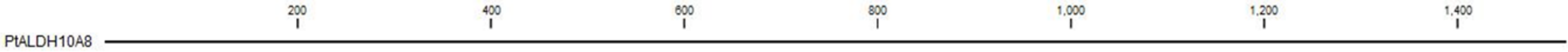

No binding site

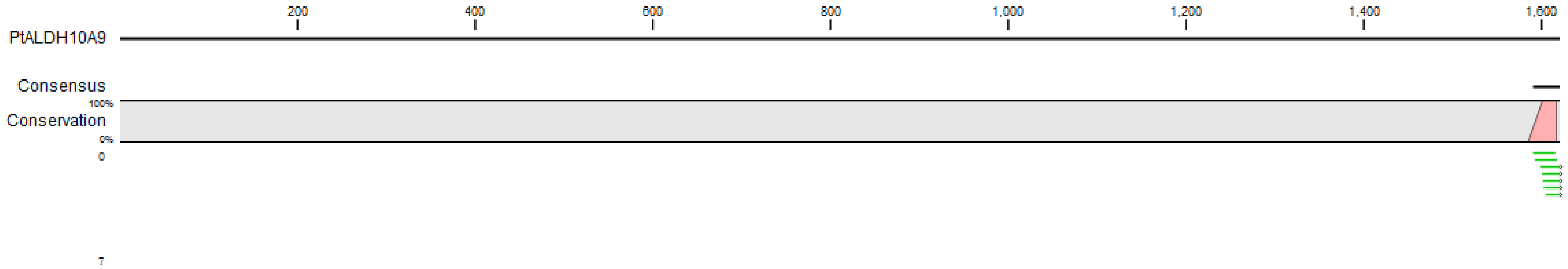

Probe sets of *PtALDH11A3:*

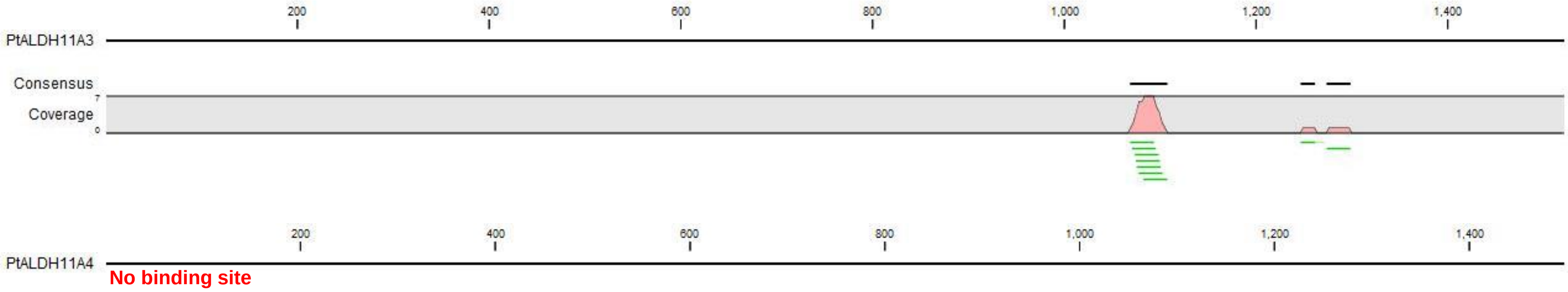

Probe sets of *PtALDH11A4:*

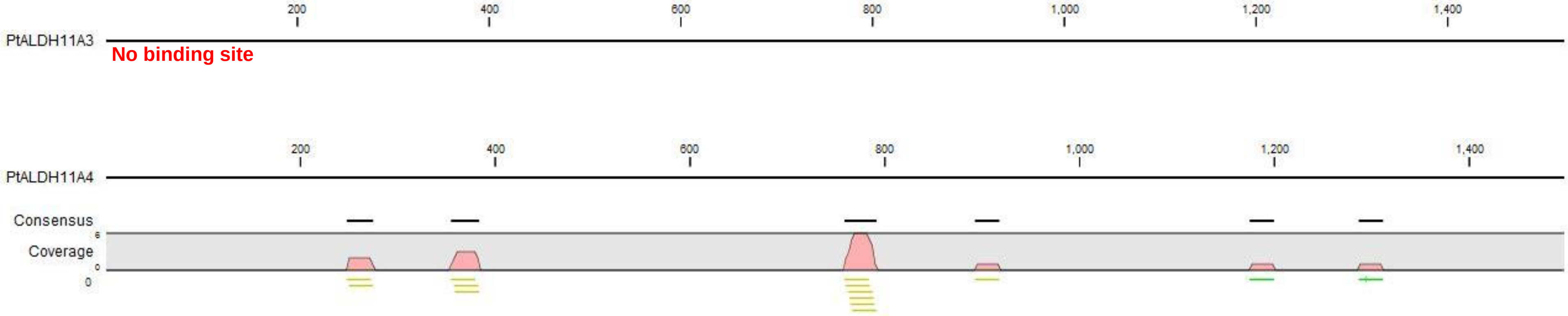

Probe sets of *PtALDH18B1:*

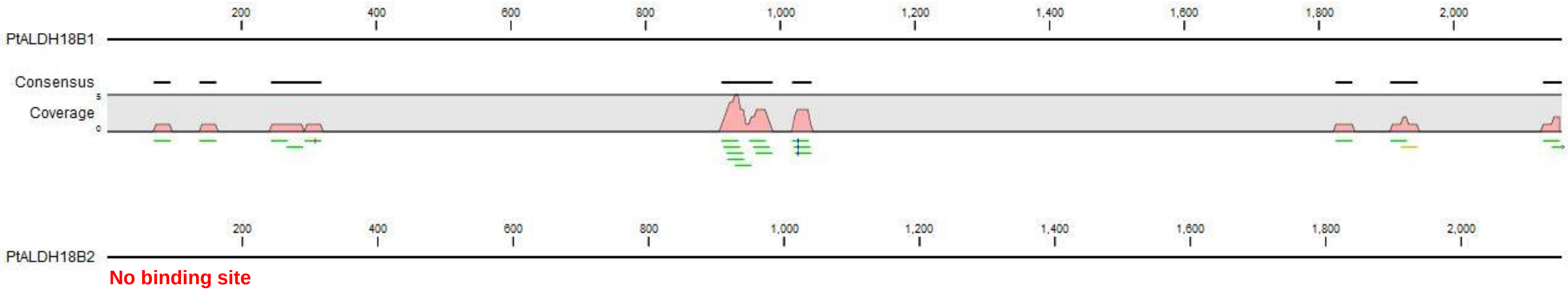

Probe sets of *PtALDH18B2:*

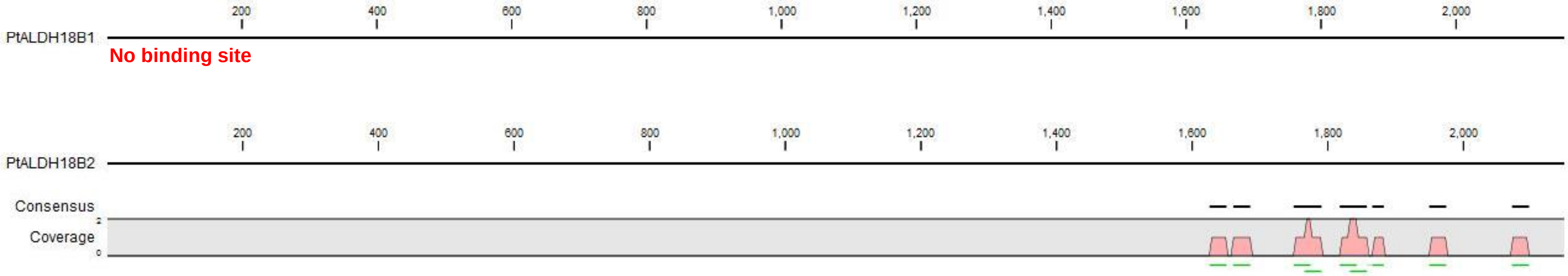

Supplement: S1 Fig — (PDF) [file pone.0124669.s001.pdf]
