# Supplementary material for: Aldehyde Dehydrogenase Gene Superfamily in Populus: Organization and Expression Divergence between Paralogous Gene Pairs
Source: PLoS One. 2015 Apr 24;10(4):e0124669. doi: 10.1371/journal.pone.0124669 (PMC4409362; doi:10.1371/journal.pone.0124669)
Supplement: S3 Table — (DOCX) [file pone.0124669.s004.docx]

## Table S3. The sequences of qRT-PCR primers.

|  | qRT-F | qRT-R |
| --- | --- | --- |
| *PtALDH2B4* | ATGGACTAACAGTTCCTGCTGACA | GCTGCATGGAGAGCAGTCAAA |
| *PtALDH2B6* | GGACTAACAGTTCCAGCGGATG | AGCTTTGCTGCATAGAGAGCAGT |
| *PtALDH3H1* | ACTCTGCATCTTGCAGTTCATAGTG | ATCACCTACAAAACTTCGATACACG |
| *PtALDH3H4* | GCTATGCATCTTGCAATTCATACC | TCGCCCATAAAACTTCGATATAGA |
| *PtALDH3H5* | AAACCTGTCATTGGGGCTATCA | CACTTCTGATCAAGCAATGCAGTT |
| *PtALDH3H6* | CAATTGATCCAGTAATAGGAGCTATTG | TTCTGCTCAAGTAACGCAGCC |
| *PtALDH6B3* | CTGTTGCAGTTACATGTGGCAAT | CTAATGTCGTCGTCGTCACAGATA |
| *PtALDH6B4* | CCTGTTGCTGTAACATGTGGTAAC | TCTAATGTCGTCGTCATCACAAATT |
| *PtALDH6B5* | TCGCCAGATTCTGAAGCGA | AAGGCTCAGCAATTGAGAGATGT |
| *PtALDH6B7* | CGACAAGTGCTTAAAGCTCTCAAG | CCGGTTGAAGATGCACTAGTGT |
| *PtALDH7B4* | TTCGAAGGTGGGTCTAATGGTC | CAACAGCAGCAAACATAACAGAAC |
| *PtALDH7B5* | TCAAAGGTGGGCCTAATGTTG | CAGCAGCAAACAGAACCGAAT |
| *PtALDH10A8* | AATTCATTGCAACCGCCAA | GCATGGAGGTTGTTACATCAATG |
| *PtALDH10A9* | AAGTATTAGAATTCATTGCAACAGCTAG | GCATGGAGGTCGTTACATCAGTA |
| *PtALDH11A3* | GGAACTAGCCAAAAGTGCACAG | TGACCTCACAACCTCAGTGACC |
| *PtALDH11A4* | ATCAGCCAAGAGCGCACAA | CCTGACCTCACAACCTCAGTTACT |
| *PtALDH18B1* | CTGATGGAATTTGTCATGTGTACA | CTCATTAAGCCCAGCGGTC |
| *PtALDH18B2* | GATGGCATTTGTCATGTCTATGTC | AGCTCATTGAGCCCACCACTA |
| *PtActin* | GTGCTTCTAAGTTCCGAACAGTGC | GACTACCAAAGTGTCTGACCACCA |
| *PtTubulin* | GATTTGTCCCTCGCGCTGT | TCGGTATAATGACCCTTGGCC |
