# Supplementary material for: Aldehyde Dehydrogenase Gene Superfamily in Populus: Organization and Expression Divergence between Paralogous Gene Pairs
Source: PLoS One. 2015 Apr 24;10(4):e0124669. doi: 10.1371/journal.pone.0124669 (PMC4409362; doi:10.1371/journal.pone.0124669)
Supplement: S5 Table — (DOCX) [file pone.0124669.s006.docx]

## Supplementary Table S5. Number of *ALDH* family members identified in various organisms.

| Organism | ALDH family | | | |  |  |  |  |  |  |  |  |  |  |  |  |  |  |  |  |  |  |  |  |  |
| --- | --- | --- | --- | --- | --- | --- | --- | --- | --- | --- | --- | --- | --- | --- | --- | --- | --- | --- | --- | --- | --- | --- | --- | --- | --- |
|  | **1** | **2** | **3** | **4** | **5** | **6** | **7** | **8** | **9** | **10** | **11** | **12** | **13** | **14** | **15** | **16** | **17** | **18** | **19** | **20** | **21** | **22** | **23** | **24** | **All** |
| *P. trichocarpa* | - | 4 | 6 | - | 1 | 4 | 2 | - | - | 2 | 3 | 1 | - | - | - | - | - | 2 | - | - | - | 1 | - | - | 26 |
| *V. vinifera* | - | 3 | 4 | - | 3 | 3 | 2 | - | - | 2 | 2 | 1 | - | - | - | - | - | 2 | - | - | - | 1 | - | - | 23 |
| *A. thaliana* | - | 3 | 3 | - | 1 | 1 | 1 | - | - | 2 | 1 | 1 | - | - | - | - | - | 2 | - | - | - | 1 | - | - | 16 |
| *Z. mays* | - | 6 | 5 | - | 2 | 1 | 1 | - | - | 3 | 1 | 1 | - | - | - | - | - | 3 | - | - | - | 1 | - | - | 24 |
| *O. sativa* | - | 5 | 5 | - | 1 | 1 | 1 | - | - | 2 | 1 | 2 | - | - | - | - | - | 2 | - | - | - | 1 | - | - | 21 |
| *P. patens* | - | 2 | 5 | - | 2 | 1 | 1 | - | - | 1 | 5 | 1 | - | - | - | - | - | - | - | - | 1 | - | 1 | - | 20 |
| *C. reinhardtii* | - | 1 | - | - | 2 | 1 | - | - | - | 1 | 1 | 1 | - | - | - | - | - | - | - | - | - | - | - | 1 | 8 |
| *O. tauri* | - | - | 1 | - | 1 | - | - | - | - | 1 | 1 | 1 | - | - | - | - | - | - | - | - | - | 1 | - | - | 6 |
| *H. sapiens* | 6 | 1 | 4 | 1 | 1 | 1 | 1 | 1 | 1 | - | - | - | - | - | - | 1 | - | 1 | - | - | - | - | - | - | 19 |
| *M. musculus* | 7 | 1 | 4 | 1 | 1 | 1 | 1 | 1 | 1 | - | - | - | - | - | - | 1 | - | 1 | - | - | - | - | - | - | 20 |
| *R. norvegicus* | 7 | 1 | 4 | 2 | 1 | 1 | 1 | 1 | 1 | - | - | - | - | - | - | 1 | - | 1 | - | - | - | - | - | - | 21 |
| *Fungi* | + | - | - | + | + | - | - | - | - | + | - | - | - | + | + | + | - | + | - | - | - | - | - | - |  |
| + and - represent presence and absence, respectively, of the *ALDH* gene family in corresponding organisms. | | | | | | | | | | | | | | | | | | | | | | | | | |
| The table modified based on Zhang et al. (2012) and Brocker et al. (2013). | | | | | | | | | | | | | | | | | | | | | | | | | |
